# A CULTURALLY-TAILORED INTERVENTION INFORMED BY CAREGIVER VOICES

**DOI:** 10.1093/geroni/igad104.1757

**Published:** 2023-12-21

**Authors:** Susanna Mage, Farnaz Hazany, Niki Cohanim, Donna Benton

**Affiliations:** University of Southern California, Los Angeles, California, United States; Sinai Adult Day Health Care Center, Los Angeles, California, United States; Sinai Adult Day Health Care Center, Los Angeles, California, United States; University of Southern California, Los Angeles, California, United States

## Abstract

The first wave of Iranian immigration to the United States occurred between 1950-1979. Now, as members of this cohort enter their 60’s, the risk of age-related dementia will increase, necessitating younger, bicultural/bilingual members of the community, often family, to take on caregiving roles. Iranian caregivers are aware of neglecting their own health due to their role and often feel sad and lonely. Despite this, they are reluctant to access community resources intended to support caregivers. This could be attributed to the desire to protect and preserve the expected family image: by not using services, the family can maintain a sense of normalcy. The Ghofetegou Koneem for Caring Families Intervention helps caregivers in Los Angeles -- home of the largest Iranian community outside of Iran -- taking care of a person with dementia. The goal is to build a network of support that is personalized, easily accessible, and addresses perceptions of available social services. To tailor the intervention for an Iranian population, investigators partnered with a local adult day health care center to conduct 1:1 interviews with caregivers and perform needs assessments to identify needs prior to development. Qualitative analysis guided by a thematic approach revealed three themes of greatest need: 1) respite/self-care, 2) advocacy, and 3) disease-specific education and training. The importance of helping caregivers self-identify was also stressed, as this may lead to increased comfort in accessing resources. Integrating the voices of Iranian caregivers will help develop a culturally-relevant intervention tailored to the needs of Iranian families.

